# INTRODUCTION

**DOI:** 10.1017/jme.2022.82

**Published:** 2022

**Authors:** Admiral Rachel L. Levine

**Affiliations:** 1. U.S. DEPARTMENT OF HEALTH AND HUMAN SERVICES, WASHINGTON, DC, USA.

**Keywords:** Transgender, Gender Non-Binary, Gender Minorities, Gender Equity, Health Equity

## Abstract

I am pleased to introduce this Symposium Edition of *The Journal of Law, Medicine & Ethics*, which covers a wide variety of issues central to transgender health equity, including Dr. Jamison Green’s recent history of the impact of health policy on transgender communities, Dr. M. Killian Kinney, Ms. Taylor Pearson, and Prof. Julie Ralston Aoki’s transgender equity tool for legal policy analysis, and Prof. Elizabeth Kukura’s analysis of issues facing transgender, non-binary, and gender expansive people during pregnancy and childbirth.

I am pleased to introduce this Symposium Edition of *The Journal of Law, Medicine & Ethics*, which covers a wide variety of issues central to transgender health equity, including Dr. Jamison Green’s recent history of the impact of health policy on transgender communities, Dr. M. Killian Kinney, Ms. Taylor Pearson, and Prof. Julie Ralston Aoki’s transgender equity tool for legal policy analysis, and Prof. Elizabeth Kukura’s analysis of issues facing transgender, non-binary, and gender expansive people during pregnancy and childbirth.

As the 17th Assistant Secretary for Health for the U.S. Department of Health and Human Services, my role is to oversee key public health offices and programs, Presidential and Secretarial advisory committees, 10 regional health offices across the country, and the Office of the Surgeon General and the US Public Health Service Commissioned Corps. I work every day to build a stronger foundation for a healthier future for all Americans. The health and well-being of transgender Americans is of particular importance to me not only because I am the highest ranking openly transgender official in the nation, but because transgender people face substantial health disparities and unique challenges in accessing appropriate healthcare.

I hope that readers of this issue come away with an understanding that transgender health is public health, and that public health includes transgender health. Estimates vary, but between 1-2% of Americans are transgender,^1^ which means the trans population is roughly the size of Los Angeles, the second-largest city in the nation.^2^ As the result of stigma and discrimination, trans people face health disparities such as disproportionately high rates of substance use disorders, mental illness, sexual and physical violence, and sexually transmitted infections.^3^ In healthcare settings, transgender patients experience high rates of discrimination, problems with insurance refusing to cover medically necessary care, and avoidance of medical care due to fear of being mistreated because of their gender identity.^4^

Transgender health is also under attack; in the 2022 legislative session, 33 state legislatures have proposed 168 new bills that would restrict access to care and involvement in public life of transgender youth and adults.^5^ Public health leaders must stand with transgender people and their allies against these unjust infringements on trans civil rights. We know that even discussion of these anti-transgender bills has already had serious, negative effects on the well-being of transgender people, and specifically has driven an increase of suicidality among transgender youth, with even worse rates among non-White youth.^6^ We must act now to ensure that transgender Americans have access to medically necessary healthcare and the support and resources to live free from discrimination.

As the 17th Assistant Secretary for Health for the U.S. Department of Health and Human Services, my role is to oversee key public health offices and programs, Presidential and Secretarial advisory committees, 10 regional health offices across the country, and the Office of the Surgeon General and the US Public Health Service Commissioned Corps. I work every day to build a stronger foundation for a healthier future for all Americans. The health and well-being of transgender Americans is of particular importance to me not only because I am the highest ranking openly transgender official in the nation, but because transgender people face substantial health disparities and unique challenges in accessing appropriate healthcare.

These are not unique public health challenges, but they will require capacity-building within public health research and practice to tackle. For many healthcare providers and public health practitioners, transgender health is a new area of practice. If you are among this group, I challenge you to read these articles with an open mind, to develop new relationships with practitioners with expertise in trans health, and to become part of the solution to the challenging problem of transgender health equity. For both readers who are new to this area of public health practice and those who have practiced in this domain for years, I hope that the articles in this Symposium Edition help you to see transgender health as central to the goals of your work.

## Note

The author has no conflicts of interest to disclose.
